# The metabolic slowdown caused by the deletion of *pspA* accelerates protein aggregation during stationary phase facilitating antibiotic persistence

**DOI:** 10.1128/aac.00937-23

**Published:** 2024-01-03

**Authors:** Yingxing Li, Xiao Chen, Weili Zhang, Kefan Fang, Jingjing Tian, Fangyuan Li, Mingfei Han, Jingjing Huang, Tianshu Sun, Fan Bai, Mei Cheng, Yingchun Xu

**Affiliations:** 1 Department of Clinical Laboratory, State Key Laboratory of Complex Severe and Rare Diseases, Peking Union Medical College Hospital, Chinese Academy of Medical Sciences and Peking Union Medical College, Beijing, China; 2 Biomedical Engineering Facility of National Infrastructures for Translational Medicine, Peking Union Medical College Hospital, Chinese Academy of Medical Sciences and Peking Union Medical College, Beijing, China; 3 Biomedical Pioneering Innovation Centre (BIOPIC), School of Life Sciences, Peking University, Beijing, China; 4 Center for Infectious Disease Research, School of Medicine, Tsinghua University, Beijing, China; 5 Clinical Biobank, Peking Union Medical College Hospital, Chinese Academy of Medical Sciences and Peking Union Medical College, Beijing, China; 6 National Center for Protein Sciences (Beijing), Beijing Proteome Research Center, Beijing Institute of Lifeomics, Beijing, China; 7 Department of Clinical Laboratory, The Affiliated Huai'an No. 1 People's Hospital of Nanjing Medical University, Huai'an, China; 8 Department of Clinical Laboratory, Jiangsu Cancer Hospital & Jiangsu Institute of Cancer Research & the Affiliated Cancer Hospital of Nanjing Medical University, Nanjing, China; Bill & Melinda Gates Medical Research Institute, Cambridge, Massachusetts, USA

**Keywords:** antibiotic persistence, persisters, dormancy depth, *pspA*, protein aggregation, VBNC cells

## Abstract

Entering a dormant state is a prevailing mechanism used by bacterial cells to transiently evade antibiotic attacks and become persisters. The dynamic progression of bacterial dormancy depths driven by protein aggregation has been found to be critical for antibiotic persistence in recent years. However, our current understanding of the endogenous genes that affects dormancy depth remains limited. Here, we discovered a novel role of phage shock protein A (*pspA*) gene in modulating bacterial dormancy depth. Deletion of *pspA* of *Escherichia coli* resulted in increased bacterial dormancy depths and prolonged lag times for resuscitation during the stationary phase. *∆pspA* exhibited a higher persister ratio compared to the wild type when challenged with various antibiotics. Microscopic images revealed that *∆pspA* showed accelerated formation of protein aggresomes, which were collections of endogenous protein aggregates. Time-lapse imaging established the positive correlation between protein aggregation and antibiotic persistence of *∆pspA* at the single-cell level. To investigate the molecular mechanism underlying accelerated protein aggregation, we performed transcriptome profiling and found the increased abundance of chaperons and a general metabolic slowdown in the absence of *pspA*. Consistent with the transcriptomic results, the *∆pspA* strain showed a decreased cellular ATP level, which could be rescued by glucose supplementation. Then, we verified that replenishment of cellular ATP levels by adding glucose could inhibit protein aggregation and reduce persister formation in *∆pspA*. This study highlights the novel role of *pspA* in maintaining proteostasis, regulating dormancy depth, and affecting antibiotic persistence during stationary phase.

## INTRODUCTION

The bacterial antibiotic persistence phenotype refers to a subgroup of cells that can transiently survive lethal antibiotic treatment and can be resuscitated to proliferate after antibiotic removal due to phenotypic heterogeneity (1, 2). Bacterial persisters are associated with prolonged infection and infection relapse (3). Previous works have also shown that bacterial persisters can accelerate the development of antibiotic resistance (4–9).

Diverse molecular mechanisms have been found to be involved in persister formation. Among them, slow growth or dormancy may be the prevailing one, because the lethal effects of antibiotics are disabled in dormant bacterial cells (10, 11). Bacteria can be induced to enter a dormant state under various conditions. For instance, starvation can induce the stringent response, activating the alarmone molecule (p)ppGpp. Subsequently, (p)ppGpp can increase the levels of type I toxin HokB by upregulating the transcriptional activator Obg. This, in turn, leads to membrane depolarization and cell dormancy (12). Similarly, DNA damage can trigger the SOS response to activate RecA, leading to the upregulation of type I toxin TisB and driving cells into dormancy (13). However, bacterial persisters always originate from cells with an appropriate dormancy depth, as cells in extremely deep dormancy become viable but non-culturable cells (VBNC cells) (14). VBNC cells cannot grow on the routine laboratory media, but they retain specific features of viable cells and can be revived under appropriate conditions (15–17). Persisters and VBNC cells represent different developmental stages of a common dormancy continuum, where persisters originate from cells in shallow dormancy (14, 16). The “dormancy depth” provides a concept to describe the degree of cellular dormant state, while the lag time can be used as the direct experimental measurement of dormancy depth. The ability to survive antibiotics is greatly improved in bacterial cells with prolonged lag times, termed “tolerance by lag” or “persistence by lag” (6, 18–20). The genetic mutations resulting in “tolerance by lag” phenotype can be fixed during the evolution of antibiotic tolerance and promote the development of antibiotic resistance in laboratory or clinical settings (5, 6, 18). Recently, an increasing number of studies have established the positive correlation between protein aggregation and bacterial dormancy depth under starvation or host cell oxidative stress (21–24). Nevertheless, our understanding of the endogenous genes that affect dormancy depth is still limited.

In this study, we found that PspA, the key effector of the phage shock protein (Psp) system, could affect the dormancy depth of *Escherichia coli* during the stationary phase. The *pspA* gene, located in the *pspABCDE* operon, is the transcriptional repressor and key effector of the Psp system (25, 26). PspA protein can counteract membrane stress and maintain proton motive force (PMF) by sealing membrane lesions and blocking proton leakage (27, 28). We uncovered that deletion of *pspA* promoted antibiotic persistence. The elevated persister ratio positively correlated with the accelerated protein aggresome formation in *∆pspA*. Finally, we identified putative underlying mechanisms through transcriptome profiling and experimental verification. We found that glucose supplementation could replenish the intracellular ATP level, prevent protein aggregation, and restore antibiotic susceptibility of *∆pspA* cells. This study shed new light on the factors influencing dormancy depth and persister formation.

## RESULTS

### *pspA* deletion increased bacterial dormancy depth and promoted persister formation in middle stationary phase

Previous studies reported that protonophore-carbonyl cyanide-m-chlorophenylhydrazone (CCCP) treatment or Obg protein overexpression can drive more cells into deeper dormancy (21, 23). Both CCCP and Obg protein can result in PMF dissipation, suggesting the potential importance of PMF maintenance in the progression of bacterial dormancy depths during the stationary phase. To verify this hypothesis, we constructed the knockout mutant of *pspA* gene, which has been proven to play a central role in PMF maintenance endogenously.

**Fig 1:**
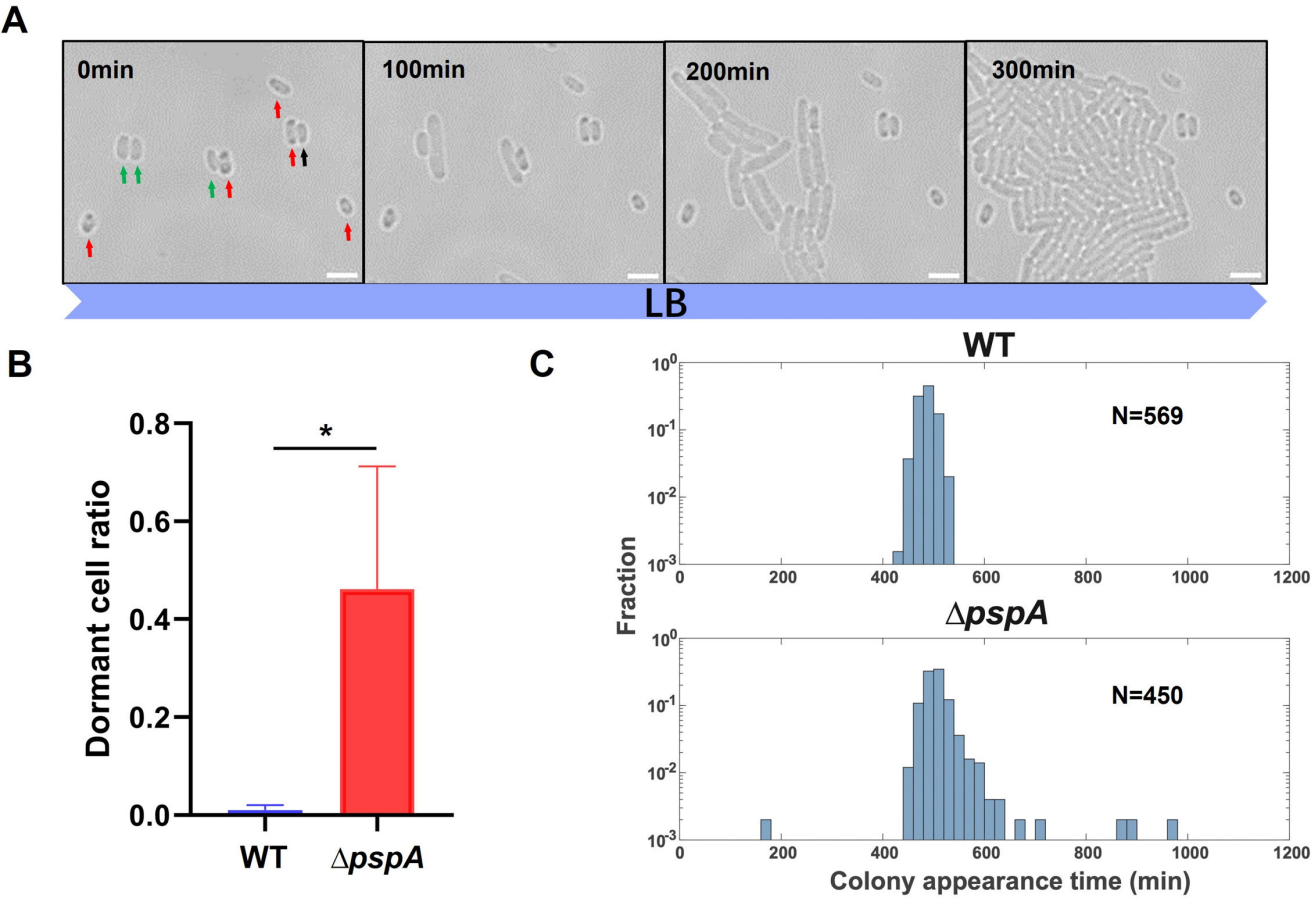
Deletion of *pspA* caused an increased dormant cell ratio and prolonged lag times. (**A**) The growth of *E. coli ∆pspA* strain from the middle stationary phase was recorded under a bright-field microscope at 37°C in LB medium. The different colors of arrows represented different cell fates during recording for 5 h. Green: active cells; Red: dormant cells; and Black: dead cells. (**B**) The dormant cell ratio of wild type and *∆pspA* calculated from time-lapse imaging. The dormant cells were defined as non-proliferating and viable cells for 5 h in fresh LB medium during imaging process. (**C**) Lag time distribution of wild type and *∆pspA* from the middle stationary phase was determined using ScanLag. The *X* axis represents the colony appearance time of samples on LB agar plates in a ScanLag setup at 37°C. N indicates the cell number measured. Scale bar, 3 µm. Each data bar in (**B**) indicates the mean ± standard deviation of at least three independent experiments. The significance of the two data bars, indicated by a line and asterisk spanning above them, was analyzed via two-tailed Student’s *t* test. *, *P* < 0.05; **, *P* < 0.01; ***, *P* < 0.005.

Consistent with our hypothesis, we found that *pspA* gene knockout mutant strain(*∆pspA*) showed increased dormancy depths in the middle stationary phase. For dormant cell ratio measurement, we monitored the regrowth process of the wild type and *∆pspA* from middle stationary phase using time-lapse imaging under a microscope at 37℃ and defined the viable and non-proliferating cells during the imaging process (for 5 h) in fresh LB medium as dormant cells. The viable cells were distinguished from the dead cells by SYTOX green staining, where SYTOX green positive cells indicated dead cells (Fig. S1A). As shown in Fig. 1A, the cells of *∆pspA* indicated by red arrows were dormant cells, and the cells indicated by green arrows were active cells. In the middle stationary phase, *∆pspA* had ~46% dormant cells, which was significantly higher than that of wild type (~1%) (Fig. 1B). However, cells that appear dormant under the microscope may have different abilities to resume growth. Some dormant cells are unable to recover from their dormant state without specific resuscitation conditions, they become VBNC cells. Only dormant cells with the ability to recover are likely to become persisters, a phenomenon called “persistence by lag” (20). Therefore, we planned to assess the resuscitation ability of dormant cells of *∆pspA* in the middle stationary phase. Lag time is the measurable quantity of the bacterial dormancy depth. Dormant cells with the ability to recover displayed prolonged lag times, while VBNC cells were not counted due to the loss of cultivability. Using the ScanLag method (29), we measured the lag time distribution of wild type and *∆pspA*. We discovered that *∆pspA* showed a wide range of lag times with a long tail in the slower recovery region, while the lag time distribution of wild type was relatively narrow (Fig. 1C). The percentage of *∆pspA* cells in the long tail position was 2% and the mean value of colony appearance time in WT and *∆pspA* population was 477.4 and 502.4 min, respectively. We excluded the possibility that the prolonged lag times of *∆pspA* were due to slower growth rates by measuring the doubling time. Fig. S1B displayed that *∆pspA* and wild type had similar growth rates. Therefore, the prolonged lag times observed in *∆pspA* were attributed to the deeper dormancy, which necessitated a longer delay to resume growth from the middle stationary phase.

**Fig 2:**
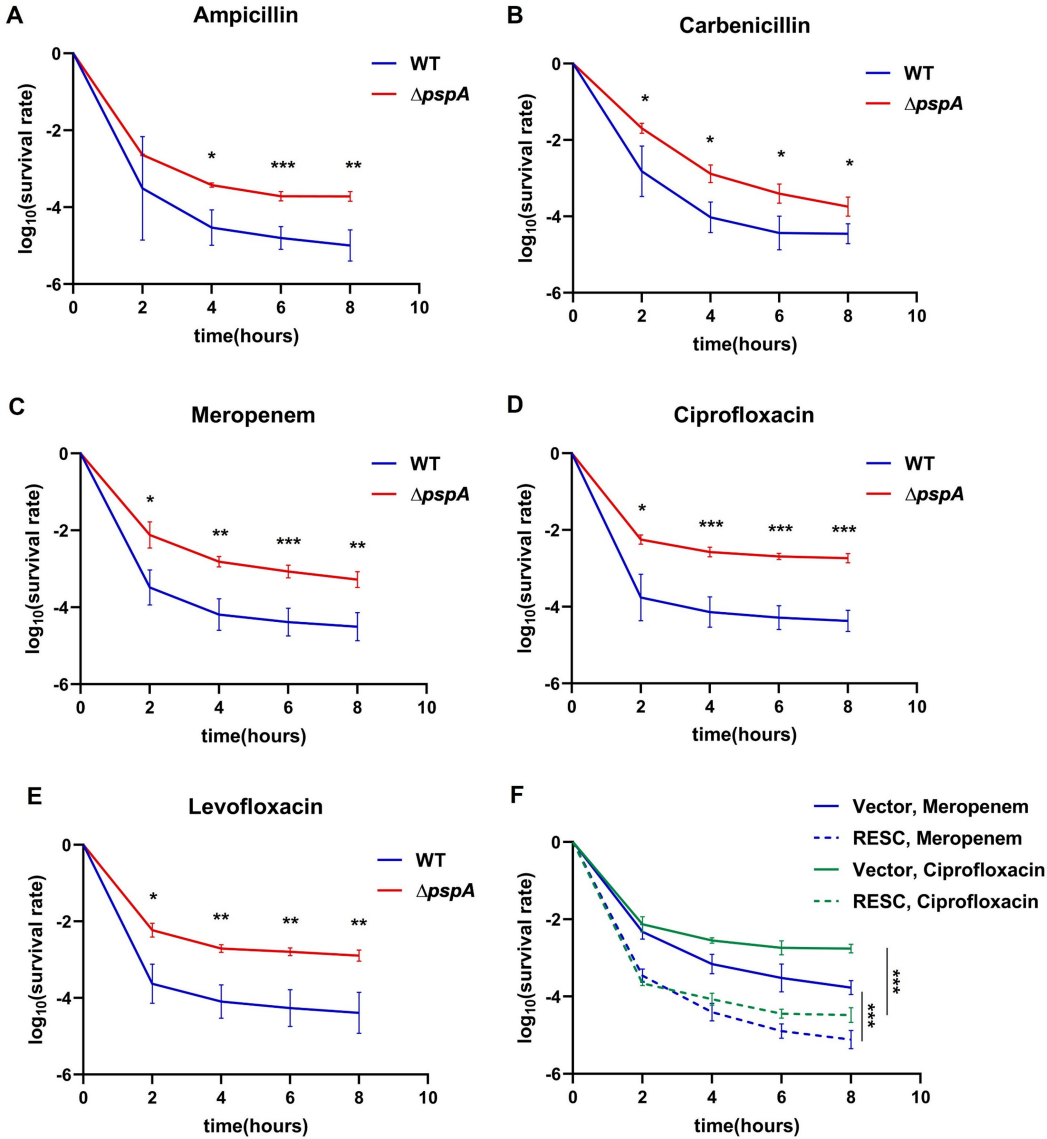
Deletion of *pspA* increased the persister ratio under various antibiotic treatments. The wild type and *∆pspA* from the middle stationary phase were diluted at a ratio of 1:20 in fresh LB medium with (**A**)100 µg/mL ampicillin, (**B**) 100 µg/mL carbenicillin, (**C**) 5 µg/mL meropenem, (**D**) 2 µg/mL ciprofloxacin, and (**E**) 5 µg/mL levofloxacin respectively, and incubated for 8 h at 37°C with shaking. (**F**) The time-kill curves of *∆pspA* with pBAD (Vector) or *∆pspA* with pBAD-*pspA* (RESC) under 5 µg/mL meropenem or 2 µg/mL ciprofloxacin for 8 h as described above. The number of viable cells was counted before and after antibiotic treatment for 2, 4, 6, and 8 h. The error bar indicates the standard deviation of at least three independent experiments. The mean survival values of wild type and *∆pspA* at the same time point (except for 0 h) were compared by two-tailed Student’s *t* test (A to E) or one-way ANOVA with Sidak’s posttest for multiple comparison (**F**). *, *P* < 0.05; **, *P* < 0.01; ***, *P* < 0.005.

To study the impact of *pspA* on antibiotic persistence, time-kill assays were conducted using different classes of antibiotics, including ampicillin, carbenicillin, meropenem, ciprofloxacin, and levofloxacin. Both wild type and *∆pspA* showed a biphasic killing where the non-persistence subpopulation (sensitive cells) died quickly in the first phase, followed by a slower decline of persistence subpopulation (persisters) in the second phase. From the time-kill curves, we determined that the persister ratio of *∆pspA* from the stationary phase was significantly higher than that of wild type under various antibiotic treatments, indicating that *pspA* deletion confers a higher antibiotic persistence phenotype (Fig. 2A through E). Moreover, *∆pspA* cells from the exponential phase exhibited a higher persister ratio compared to wild-type cells under meropenem or ciprofloxacin treatment (Fig. S2A and B). We also performed the minimal inhibitory concentration (MIC) measurement of wild type and *∆pspA* for the antibiotics used in this study. The unchanged MIC values of wild type and *∆pspA* suggested that the enhanced survival of *∆pspA* under antibiotics was not due to resistance mechanisms (Table S1). Then, we constructed the *pspA* rescued strain, *∆pspA* with pBAD-*pspA* (RESC), and compared the time-kill curves of *pspA* rescued strain with those of the control. We found that the persister ratio of *pspA* rescued strain was significantly lower than that of the control (Fig. 2F; Fig. S2C through E), confirming that the wild-type version of *pspA* could rescue the high persistence phenotype.

### Accelerated formation of intracellular protein aggresomes of *∆pspA* positively correlated with antibiotic persistence

**Fig 3:**
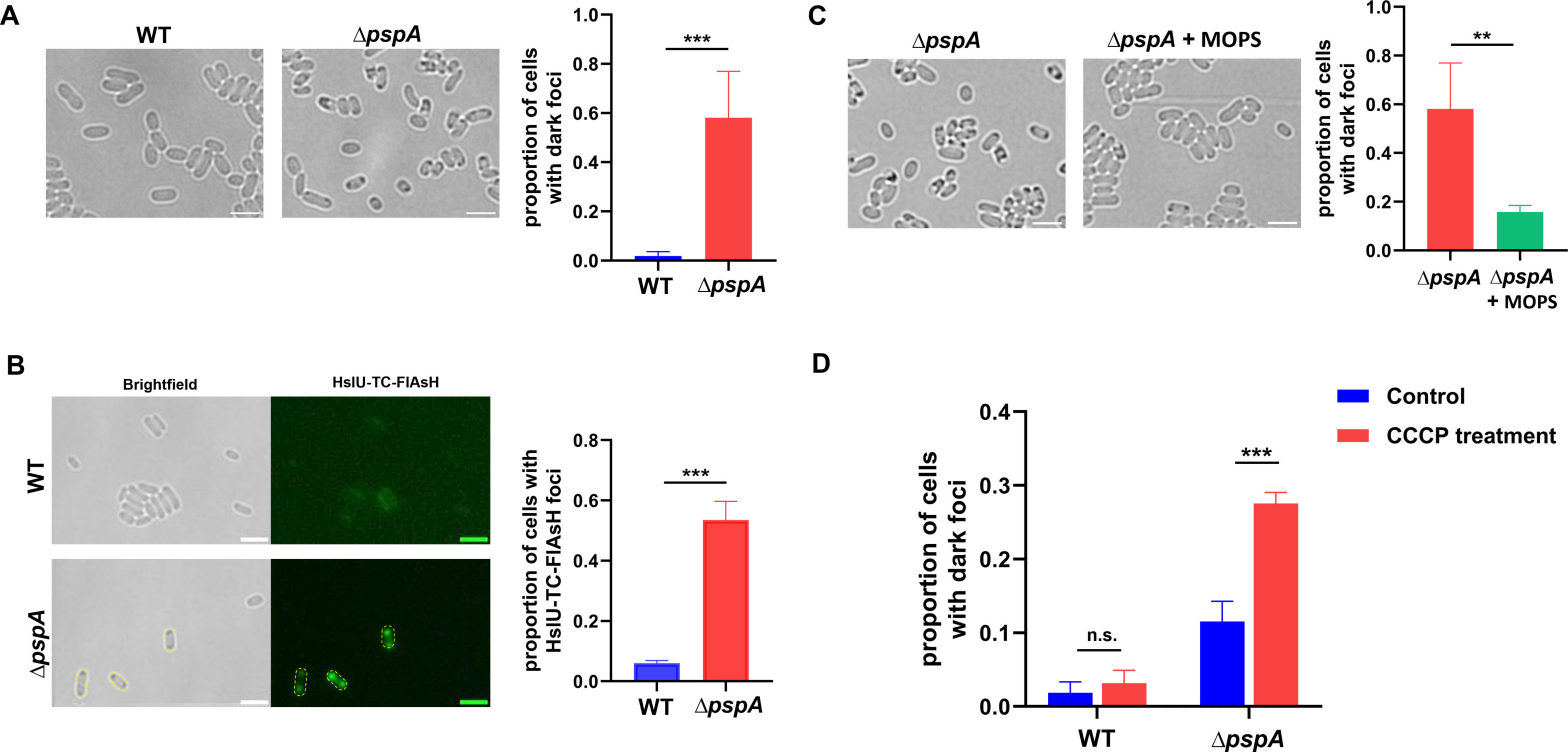
Lack of *pspA* accelerated intracellular protein aggresome formation during stationary phase. (**A**) Bright-field images of wild type and *∆pspA* from the middle stationary phase showed that *∆pspA* had more cells with dark foci. Quantification of proportion of cells with dark foci from the above bright-field images (right panel). (**B**) Bright-field and fluorescence images of HslU-TC-FlAsH labeled cells in wild type and *∆pspA* showed that *∆pspA* had more cells with HslU-TC-FlAsH fluorescence foci, which acted as the biomarker of protein aggresomes. The cells outlined by yellow dotted line displayed co-localization of HslU-TC-FlAsH fluorescence foci and dark foci within cells. Quantification of proportion of cells with HslU-TC-FlAsH fluorescence foci from the images (right panel). (**C**) Bright-field images of *∆pspA* treated with or without 40 mM MOPS from the middle stationary phase showed that MOPS prevented dark foci formation within *∆pspA* cells. Quantification of proportion of cells with dark foci from the bright-field images (right panel). (**D**) Lack of *pspA* accelerated intracellular protein aggresome formation under CCCP treatment. The wild type and *∆pspA* cells from early stationary phase were split into two tubes, one tube with 20 µM CCCP for 1.5 h, and the other tube with no CCCP for 1.5 h. The proportion of cells with dark foci was calculated from the bright-field images of different samples. Scale bar, 3 µm. Each data bar indicates the mean ± standard deviation of at least three independent experiments. The significance of the two data bars, indicated by a line and asterisks spanning above them, was analyzed via two-tailed Student’s *t* test. *, *P* < 0.05; **, *P* < 0.01; ***, *P* < 0.005.

When measuring the dormant cell ratio of *∆pspA* under a microscope, we noticed dark foci within the dormant cells in *∆pspA* (Fig. 1A). Based on previous research, the dark foci observed under the bright-field microscope are a collection of endogenous protein aggregates, which can serve as an indicator of cell dormancy (21). We then compared the ratio of cells containing dark foci between ∆*pspA* and wild type from middle stationary phase. After incubating in LB medium for 16 h, the percentage of cells with dark foci in *∆pspA* was dramatically higher than that in wild type (Fig. 3A). After quantitative analysis, we found that the ratio of cells with dark foci in *∆pspA* was approximately 58%, while it was only 1.8% in wild type (Fig. 3A). To verify the properties of dark foci appearing in *∆pspA* cells, we examined the colocalization between the biomarker of cellular protein aggresomes and the dark foci. Since the HslU protein is abundant in protein aggresomes, we used fluorescently labeled HslU protein as the biomarker of protein aggresomes according to the previous work (21). We labeled HslU with TC-FlAsH, a marker whose fluorescence increases more than threefold when protein aggregation occurs (30). In addition, we also constructed a chromosomally labeled *hslU-egfp* strain. Both HslU-TC-FlAsH and HslU-EGFP fluorescent foci colocalized with dark foci (Fig. 3B; Fig. S3). We determined the proportion of cells containing protein aggresomes labeled with HslU-TC-FlAsH in wild type and *∆pspA*. As shown in Fig. 3B, the proportion of cells with HslU-TC-FlAsH fluorescent foci in *∆pspA* was approximately 54%, which was significantly higher than that in the wild type (~6%). Likewise, *∆pspA* from the middle stationary phase displayed a higher number of cells containing HslU-EGFP fluorescent foci compared to the wild type (Fig. S3).

According to previous research, MOPS (3-N-morpholinopropanesulfonic acid) can prevent protein aggregation in late stationary phase by acting as an osmolyte or a pH buffer (31–34). To examine whether MOPS supplementation could attenuate protein aggresome formation in *∆pspA* from the middle stationary phase, we treated *∆pspA* cells in LB medium with or without 40 mM MOPS for 16 h, respectively. As expected, we observed a significant decrease in the proportion of cells with dark foci in *∆pspA* cells supplemented with MOPS, which decreased to 16% compared to the control counterpart (58%) (Fig. 3C). In addition to prolonged stationary phase culturing, CCCP could induce the appearance of dark foci. In the early stationary phase (incubating in LB medium for 12 h), cells treated with 5–50 μM CCCP for 4 h could dramatically induce the formation of protein aggresomes (21). Here, we treated cells with 20 µM CCCP for only 1.5 h, which was not long enough to induce aggresomes significantly in wild type (Fig. 3D). However, the ratio of cells with aggresomes increased notably in *∆pspA* during merely 1.5 h CCCP treatment (Fig. 3D). These results suggested that deleting *pspA* gene could accelerate protein aggresome formation under CCCP treatment. Based on previous studies suggesting a positive correlation between PMF depolarization and protein aggresome formation (21, 33), we hypothesized that the addition of CCCP would further disrupt cellular PMF in *∆pspA*. We used a membrane potential-sensitive fluorescent dye, DiBAC_4_(3), to indicate the PMF of wild type, *∆pspA,* wild type treated with CCCP and *∆pspA* treated with CCCP. DiBAC_4_(3) is an anionic probe and can enter the depolarized cells where it binds to intracellular proteins or membranes and exhibits enhanced fluorescence. Consistent with our hypothesis, Fig. S4 showed that CCCP treatment could induce the depolarization of membrane potential both in wild type and *∆pspA* cells significantly*,* but *∆pspA* cells exhibited a higher degree of membrane depolarization than wild type cells after CCCP treatment.

**Fig 4:**
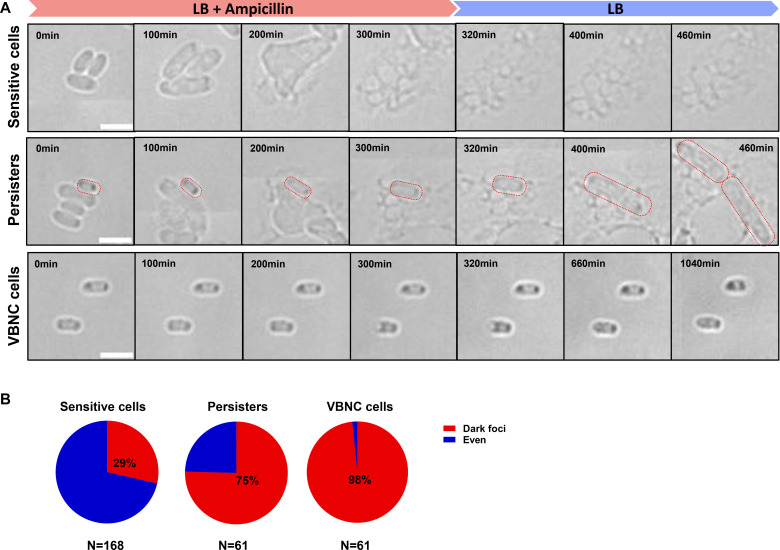
Antibiotic persistence of *∆pspA* was positively correlated with protein aggresome formation. (**A**) Time-lapse images of the antibiotic killing and recovery process of *∆pspA*. According to the different cell responses to antibiotic treatment, we categorized the whole population of *∆pspA* into three subgroups: sensitive cells, persisters, and VBNC cells. Sensitive cells were those that were killed during antibiotic treatment. Persisters were a small group of cells that survived and resuscitated after antibiotic removal, and the persister shown in the figure was outlined by a red dotted line. VBNC cells were the cells that remained in a dormant state throughout the antibiotic treatment and after its removal. (**B**) The proportion of cells with dark foci in each of the three cell subgroups of *∆pspA* (sensitive cells, persisters, and VBNC cells). N indicates the cell number measured. Scale bar, 3 µm.

To investigate the relationship between protein aggregation and antibiotic persistence in *∆pspA* at the single-cell level, we monitored the antibiotic-killing process of *∆pspA* in real time under the microscope and recorded the fates of individual cells. As shown in Fig. 4A, most cells began to proliferate and were killed by lysis, which were called sensitive cells; a small group of cells survived due to a longer lag time and resuscitated after antibiotic removal, which were called persisters; another group of viable cells remained in a dormant state throughout the antibiotic treatment and after its removal, which were called VBNC cells. Dead cells with normal morphology were excluded by SYTOX green staining. Persisters and VBNC cells are both drug-tolerant cells, but differ in their abilities to escape from dormancy. Then, we calculated the proportion of cells with dark foci in each of the three cell subgroups of *∆pspA* (sensitive cells, persisters, and VBNC cells). Our analysis revealed that 29% of sensitive cells, 75% of persister cells, and 98% of VBNC cells showed the presence of dark foci (Fig. 4B), indicating a positive correlation between protein aggregation and drug-tolerant cells in *∆pspA* at the single-cell level.

### Loss of *pspA* gene led to the collapse of cellular proteostasis and a general metabolic slowdown

**Fig 5:**
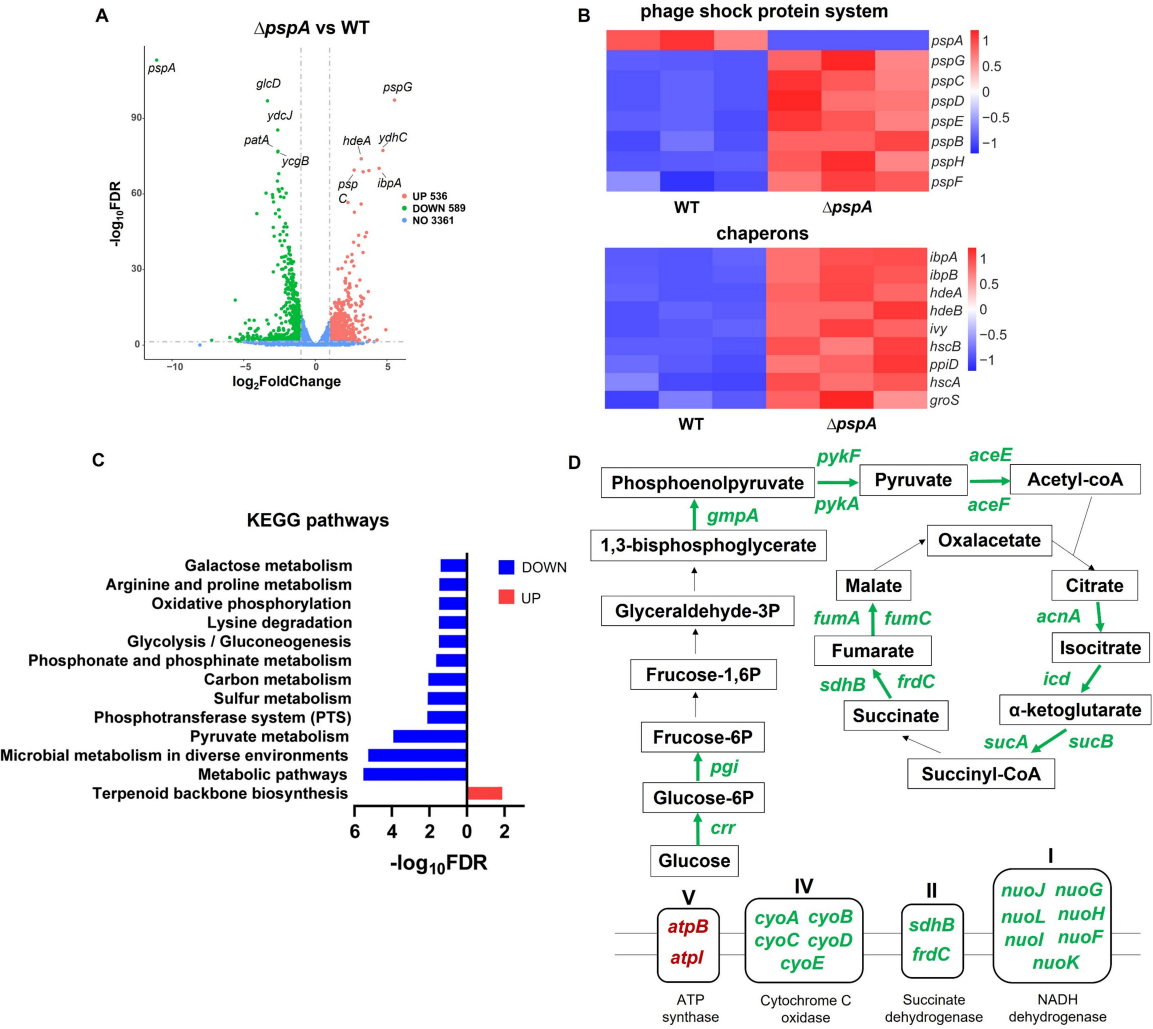
The transcriptome analysis between *∆pspA* and wild type. (**A**) Volcano plot of DEGs between *∆pspA* and wild type. The DEGs were defined on the basis of the absolute fold change >2 and the adjusted *P* value (FDR) < 0.05. (**B**) Heat maps indicated the normalized expression of DEGs belonging to phage shock protein system and chaperons in wild type and *∆pspA*. (**C**) Significantly enriched KEGG pathways of downregulated (blue) and upregulated (red) genes from DEGs. The significantly enriched KEGG pathways were defined on the basis of the adjusted *P* value (FDR) < 0.05 through DAVID website. (**D**) Upregulated and downregulated genes in the ATP-generating pathways, including glycolysis/gluconeogenesis, TCA cycle, and oxidative phosphorylation. The DEGs were labeled in the specific position of the schematic diagram according to their function. The genes with green color represented the downregulated genes in *∆pspA*, while the genes with red color represented the upregulated genes. The genes whose expression levels did not change significantly according to our criteria for DEGs were not labeled in this diagram.

To better understand the molecular mechanism underlying protein aggresome formation in *∆pspA*, we performed transcriptome profiling of wild type and *∆pspA*. RNA was extracted from wild type and *∆pspA* from the middle stationary phase and then prepared for the Illumina-based sequencing. After data analysis, we obtained a comprehensive data set including differential gene expression (Table S2). Compared to wild type, *∆pspA* from middle stationary phase had 536 upregulated genes and 589 downregulated genes [>2 × fold change, adjusted *P* value (FDR) < 0.05] (Fig. 5A). From the differential expressed genes (DEGs), we first checked the expression changes of phage shock response system. PspA can form an inhibitory complex with PspF, which is the transcriptional activator of phage shock protein (*psp*) operon. As a result, PspF becomes constitutively active in *∆pspA* (26). We found the upregulation of all other *psp* response genes in *∆pspA* strain, including *pspBCDE*, *pspG*, and *pspH* (Fig. 5B). Then, we examined the expression of genes with chaperon activities, given the accelerated appearance of protein aggresomes. As expected, we discovered the significant upregulation of genes responsible for cellular proteostasis, including *ibpA*, *ibpB*, *hdeA*, *hdeB*, *ivy*, *hscB*, *ppiD*, *hscA*, and *groS* (Fig. 5B). IbpA and IbpB can associate with aggregated proteins to protect them from denaturation during heat shock treatment (35). Ivy, HscA, HscB, and groS are involved in protein folding or maturation. HdeA, HdeB, and PpiD exhibit a chaperone-like activity in periplasmic space. This result reflected the collapse of proteostasis in *∆pspA*, consistent with the observation of protein aggresomes under the microscope.

For gene functional enrichment analysis, the differentially expressed genes (DEGs) were submitted to DAVID for KEGG pathway analysis. The KEGG items with an adjusted *P* value < 0.05 were identified from DEGs (Fig. 5C). The downregulated genes mainly enriched in metabolism-related pathways, including pyruvate metabolism, sulfur metabolism, carbon metabolism, phosphonate and phosphinate metabolism, arginine and proline metabolism, galactose metabolism, lysine degradation, and phosphotransferase system (PTS). This result indicated a possible metabolic slowdown in *∆pspA*, consistent with its deeper dormancy state. In addition, the downregulated genes also enriched in energy generating pathways, including glycolysis/gluconeogenesis and oxidative phosphorylation, suggesting that *∆pspA* cells might have a lower energy status. The upregulated genes in *∆pspA* only significantly enriched in terpenoid backbone biosynthesis pathway. Terpenoid is also known as isoprenoid, a class of organic compounds important for cell survival. Isoprenoid quinones are essential groups of compounds occurring in membranes, such as ubiquinone and menaquinone involving in electron transport (36). The modulation of isoprenoid biosynthesis affects membrane stress and antibiotic resistance in *Acinetobacter baumannii* (37).

We paid special attention to the ATP-generating pathways, as previous studies have reported that ATP depletion induces protein aggregation in bacteria (21, 23). We found that most DEGs involved in the carbon metabolism and respiration were downregulated in *∆pspA* cells (Fig. 5D). The abundance of *crr*, *pgi*, *gmpA*, *pykF*, and *pykA* involved in the glycolysis/gluconeogenesis pathway, *aceE*, and *aceF* participated in the pyruvate metabolism pathway, *acnA*, *icd*, *sucA*, *sucB*, *sdhB*, *frdC*, *fumA*, and *fumC* engaged in the TCA cycle, *nuoF-L*, and *cyoA-E* responsible for the oxidative phosphorylation all decreased in *∆pspA* (Fig. 5D). Only *atpB* and *atpI* constituting the ATP synthase were upregulated. These results indicated that the loss of *pspA* caused the general slowdown of ATP-generating activities.

In summary, as revealed by the transcriptomic data, the *pspA* deletion influenced gene expression across various aspects, suggesting the collapse of cellular proteostasis and a general metabolic slowdown in *∆pspA*, which might facilitate protein aggregation and contribute to the progression of dormancy depths.

### ATP replenishment of *∆pspA* via glucose supplementation prevented protein aggregation and alleviated antibiotic persistence

**Fig 6:**
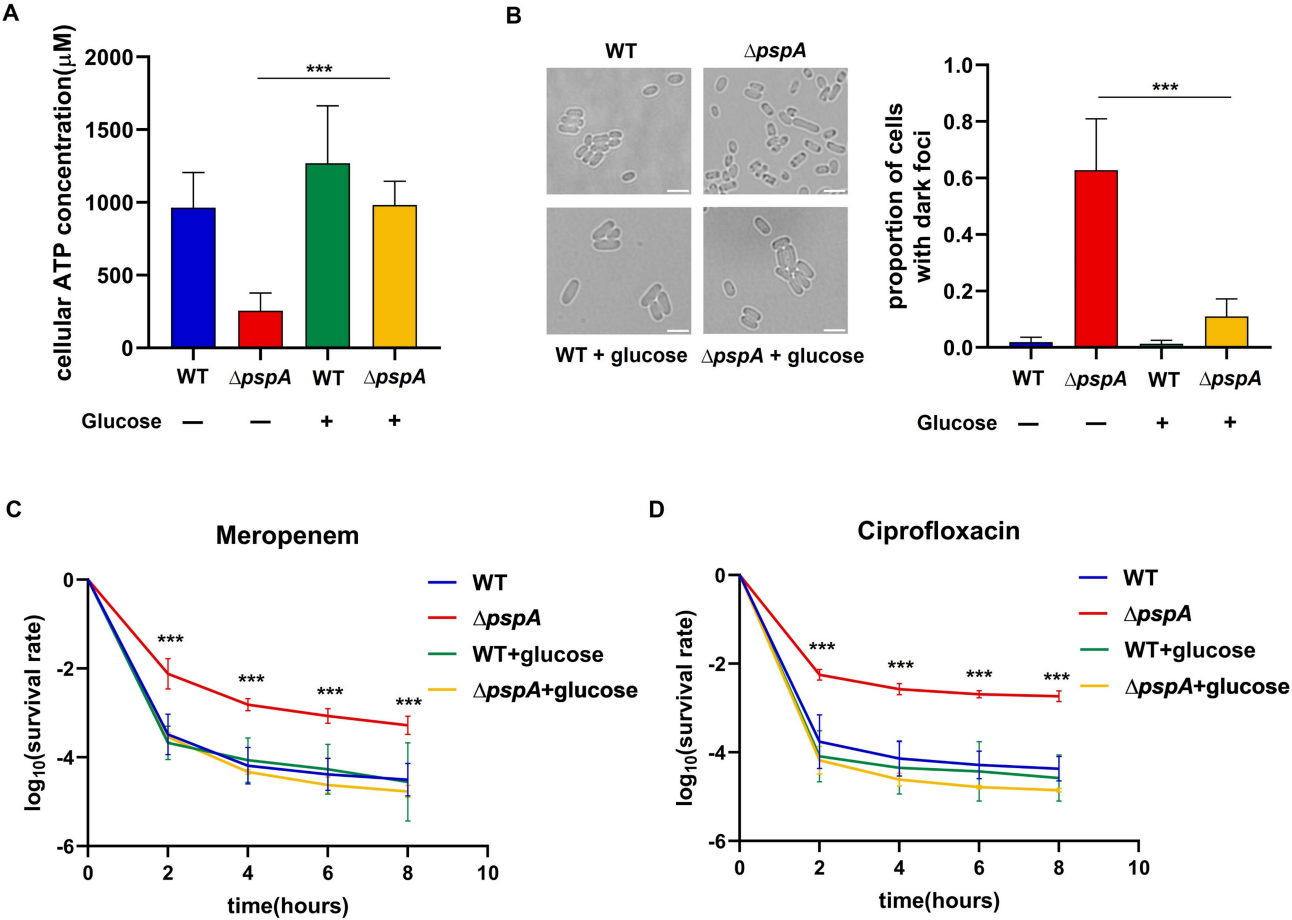
The effects of glucose supplementation on cellular ATP level, protein aggresome formation, and persister ratio in *∆pspA*. (**A**) The cellular ATP concentration of wild type, wild type supplemented with 0.5% glucose, *∆pspA*, and *∆pspA* supplemented with 0.5% glucose from the middle stationary phase was calculated. (**B**) Bright-field images of wild type, wild type supplemented with 0.5% glucose, *∆pspA*, and *∆pspA* supplemented with 0.5% glucose in the middle stationary phase. Quantification of proportion of cells with dark foci from the above bright-field images (right panel). (**C**) and (**D**) The time-kill curves of wild type, wild type supplemented with 0.5% glucose, *∆pspA*, and *∆pspA* supplemented with 0.5% glucose from the middle stationary phase under 5 µg/mL meropenem or 2 µg/mL ciprofloxacin treatment. The number of viable cells was counted before and after antibiotic treatment for 2, 4, 6, and 8 h. The survival values of wild type in (**C and D**) were duplicated from Fig. 2C and D. All data presented are means ± standard deviation of at least three independent experiments. The mean values of *∆pspA* and *∆pspA* supplemented with glucose were compared by two-tailed Student’s *t* test (**A and B**). The mean survival values of *∆pspA* and *∆pspA* supplemented with glucose at the same time point (except for 0 h) were compared by two-tailed Student’s *t* test (**C and D**). Scale bar, 3 µm. *, *P* < 0.05; **, *P* < 0.01; ***, *P* < 0.005.

Based on our transcriptomic profiling results and previous studies (21, 23), we examined the cellular ATP level of *∆pspA* by using the BacTiter-Glo assay (38). As expected, we observed that the cellular ATP concentration of *∆pspA* decreased notably than that of wild type (Fig. 6A). Given the carbon metabolism slowdown observed in *∆pspA*, we treated the *∆pspA* cells with 0.5% glucose in LB medium, which restored the ATP concentration of *∆pspA* to the wild-type level (Fig. 6A). To assess whether the ATP depletion in *∆pspA* contributed to protein aggregation, we analyzed the proportion of cells with dark foci in *∆pspA* supplemented with glucose under the microscope. As shown in Fig. 6B, the glucose supplementation significantly reduced the proportion of *∆pspA* cells with dark foci to 15% in the middle stationary phase. Next, to evaluate the effects of glucose treatment on the antibiotic persistence phenotype of *∆pspA*, we compared the time-kill curves of wild type, *∆pspA*, wild type treated with glucose and *∆pspA* treated with glucose under meropenem or ciprofloxacin treatment. The results showed that glucose supplementation decreased the persister ratio of *∆pspA* to the wild type level under both meropenem and ciprofloxacin treatment, but had no effect on the persister ratio of wild type (Fig. 6C and D). The evidence suggested that ATP depletion in *∆pspA* cells was positively associated with protein aggresome formation and persister ratio. Above all, glucose supplementation reduced protein aggregation and alleviated antibiotic persistence ability in *∆pspA* probably by modulating the cellular ATP concentration.

## DISCUSSION

In this study, we discovered that the loss of *pspA* gene promoted the formation of intracellular protein aggresomes during the stationary phase, which appeared as dark foci under bright-field microscopy. Furthermore, *∆pspA* exhibited a deeper state of dormancy and prolonged lag times, which enabled it to survive better under various antibiotics. The increased persister ratio of *∆pspA* was positively correlated with protein aggregation at the single-cell level. According to the transcriptome profiling results, we discovered the upregulation of proteostasis maintenance systems and the downregulation of metabolic and energy-generating activities. We validated that ATP depletion caused by the metabolic slowdown in *∆pspA* induced the protein aggresome formation and increased antibiotic persistence level. The replenishment of cellular ATP concentration by adding glucose disabled the protein aggresome formation and restored the antibiotic susceptibility of *∆pspA*.

Lag time is the experimentally measurable quantity of bacterial dormancy depth and lag time optimization plays an important role in the evolution of antibiotic tolerance and resistance. In the case of intermittent exposure to ampicillin, bacteria can rapidly develop a drug-tolerance phenotype through extended lag times, followed by the emergence of a drug-resistance phenotype. The genetic mutations underlying the adjustment in lag-time distribution have been found in genes involved in protein synthesis (*metG*), protein transport (*prsA*), and so on (5). Evidence has shown that mutations resulting in “tolerance by lag” were fixed in the evolved population, promoting the evolution of resistance of clinical *Staphylococcus aureus* strains in patients under antibiotic treatment (6). These genes, whose mutations cause “tolerance by lag” phenotype, are defined as *tbl* genes (18). According to this definition, *pspA* can be considered as a “persistence by lag” gene. We speculate that if the function-deficient mutations occur in *pspA* gene, the mutant will have a survival advantage under antibiotic treatment and is more likely to develop drug resistance.

The appearance of protein aggresomes means the collapse of proteostasis. The transcriptome analysis also showed the upregulation of proteostasis maintenance genes in *∆pspA*, including *ibpA*, *ibpB*, *hdeA*, *hdeB*, *ivy*, *hscA*, *hscB*, *ppiD*, and *groS*, which assisted in protein folding or maturation. Many studies have proven the link between protein aggregation and persister formation (22, 23, 39, 40). Protein aggregation is proposed to drive bacterial cells into deeper dormancy by sequestering proteins with essential physiological functions. Dormant cells may require specific cellular machinery to eliminate the protein aggresome to resume growth, leading to the lag time elongation. There are many conditions that can trigger protein aggregation. In *∆pspA*, we found that ATP depletion contributed to the protein aggresome formation. Previous studies have demonstrated that ATP acts as a biological hydrotrope to maintain protein solubility in a crowded cytoplasm, soluble proteins are more likely to precipitate when cellular ATP decreases (41, 42), and ATP depletion leads to protein aggresome formation by liquid-liquid phase separation (33). In addition, *pspA* is required for the efficient protein translocation across the inner membrane (43, 44). The impaired protein translocation of *∆pspA* may lead to the abnormal accumulation of periplasmic or outer membrane proteins in the cytoplasm or inner membrane, triggering the protein aggregation of *∆pspA*. However, we should also be cautious about another possibility. The conditions that can trigger protein aggregation, such as ATP depletion, can promote antibiotic persistence directly or by other means independent of protein aggregation. Therefore, the possibility that protein aggregation and antibiotic persistence only have a co-incidental rather than a causative relationship cannot be ruled out.

As the key effector of phage shock response, PspA protein is strictly conserved across bacterial phyla and is critical for membrane protection and remodeling (45). PspA belongs to the ancient Endosomal Sorting Complexes Required for Transport (ESCRT)-III membrane-remodeling superfamily through evolutionary and structural analyses (27, 46). The expression of *pspA* is strongly induced by various factors affecting cytoplasmic membrane function or integrity. In stationary phase, bacteria devote a significant share of their limited protein synthesis resources to PspA production (26, 47). All of the evidence points to the importance of PspA protein in bacterial physiological maintenance. Nonetheless, the understanding of the phenotype of *pspA*-lacking bacterial cells is still limited. In this study, we first revealed the relationship between *pspA* and cellular dormancy depths. Our experiment proved that *∆pspA* cells obtained deeper dormancy than wild type probably driven by protein aggregation, leading to the increase of persisters. Recent studies have demonstrated that proton-ionophore CCCP, type I toxin-HokB that can punch holes in cell membranes, sub-lethal doses of antimicrobial peptides priming, or stimulation of host defense peptide LL-37 in serum can promote bacterial survival in the presence of antibiotics (21, 33, 48, 49). We can infer that factors causing cell membrane damage or PMF dissipation can increase bacterial population fitness to antibiotics as a bet-hedging strategy. The phenotype of *∆pspA* in this study provided robust support for this inference.

The observed ATP depletion and possible metabolic slowdown suggested by transcriptome profiling in *∆pspA* cells were most likely due to the impaired membrane protection function of *∆pspA*. In *E. coli*, the cells that lack the outer membrane channel TolC, the increased amount of PspA and metabolic shutdown were observed, especially when entering the stationary phase, hinting on the link between the membrane stress and cellular metabolic state (50). PspA protein might prevent proton leakage to maintain PMF, and the dissipation of PMF was observed in *∆pspA* (26, 45). The dissipation of PMF in *∆pspA* might trigger the metabolic slowdown since the ATP synthesis through oxidative phosphorylation was perturbed. However, it appears that the possible metabolic slowdown of *∆pspA* is specific to the stationary phase, as *∆pspA* cells in the exponential phase show similar doubling time with the wild type. This is likely because *pspA* responds to the membrane stress and plays an effective membrane-repairing role in the stationary phase, which is not required in the exponential phase.

There is an interesting phenomenon regarding the role of *pspA* gene in antibiotic evasion. Recently, Wang et al. reported that *pspA* played a positive role in the starvation-induced antibiotic tolerance, and deletion of *pspA* compromised this tolerance by reducing efflux activity (51). However, in our study, *pspA* gene had an opposite effect on antibiotic tolerance under a different experimental condition. This discrepancy can be attributed to the difference in the nutrient environment during antibiotic-killing process. In the nutrient-rich condition where bacteria can proliferate, the slow exit from the dormant state of *∆pspA* protects it from the lethal effects of various antibiotics that target growing bacteria. Nevertheless, in the nutrient-depletion condition where all cells are in a dormant phase, the reduction in PMF maintenance and efflux activity of *∆pspA* diminishes its ability to survive the slow antibiotic-killing process (144 h) (51). We conjecture that in the acute phase of bacterial infection, the dysfunction of *pspA* promotes antibiotic evasion, while in the persistence phase of bacterial infection, the dysfunction of *pspA* inhibits the long-term survival in the presence of antibiotics. This interesting phenomenon also reminds us that the mechanism of antibiotic evasion may vary greatly during different stages of infection, and even the same gene (such as *pspA*) may play completely opposite roles in different stages of infection.

Our study shed new light on the role of *pspA* gene in bacterial protein aggresome formation and antibiotic persistence. Further investigating the intriguing relationship between phage shock response and protein homeostasis in *∆pspA* may yield new ideas for combating bacterial infections.

## MATERIALS AND METHODS

### Bacterial strains and growth conditions

The bacterial strains used in our study are listed in Table 1. *E. coli* strains BW25993, BW25993 *hslU- TCtag*, and BW25993 *hslU-egfp* were grown in Luria Broth (LB; Sangon Biotech); BW25993 *∆pspA*, BW25993 *hslU-TCtag ∆pspA* and BW25993 *hslU-egfp ∆pspA* were cultured in LB with 50 µg/mL kanamycin; BW25993 *∆pspA* with pBAD (Vector) and BW25993 *∆pspA* with pBAD-*pspA* (RESC) were cultured in LB with 50 µg/mL kanamycin and 25 µg/mL chloramphenicol. Early stationary, middle stationary, or late stationary samples were obtained by incubating the specific strains in LB medium for 12, 16, or 24 h, respectively, with shaking (220 rpm) at 37°C. BW25993 was used as the wild-type strain, unless specifically noted otherwise.

**TABLE 1:** Bacterial strains and plasmids used in this study

Bacterial strain	Source	Identifier/Reference
BW25993	Yale Coli Genetic Stock Center	CGSC#: 7639
BW25993 *∆pspA*	This study	N/A
BW25993 *hslU- TCtag*	Bai Lab	(21)
BW25993 *hslU- TCtag ∆pspA*	This study	N/A
BW25993 *hslU- egfp*	This study	N/A
BW25993 *hslU- egfp ∆pspA*	This study	N/A
BW25993 *∆pspA* with pBAD	This study	N/A
BW25993 *∆pspA* with pBAD-*pspA*	This study	N/A
Plasmid	Description	Source
pSIM6	helper plasmid for homologous recombination, Ampicillin	(52, 53)
pCP20	helper plasmid for Flp recombinase-mediated excision, Ampicillin/chloramphenicol	(54)
pBAD/Myc-His A	Expression plasmid replaced with chloramphenicol resistance cassette, Chloramphenicol	(11)

### Bacterial strain construction

Strains containing *pspA* gene deletion and chromosomal *hslU-egfp* fusion were constructed by λ-Red mediated homologous recombination (54–56). For *hslU-egfp* strain construction, we first amplified the *egfp* DNA fragment and linked it with the kanamycin-resistance cassette flanked by FRT sites by overlap PCR. Then, the fragment was amplified using the long primer containing homology arm complementary to the flanking sequence of the insertion site at the 3′ end of *hslU* gene on the chromosome, which was used as the template for homologous recombination. Then, the template was transformed into electrocompetent cells with an induced recombineering helper plasmid (pSIM6) (52, 53). After 5 h of recovery, transformed cells were plated on a selection plate containing kanamycin and incubated at 30°C overnight. The positive clones were selected and verified by PCR. Cells of the corrected clone were cultured in LB medium with shaking (220 rpm) at 37°C to remove the pSIM6 plasmid. Next, we transformed the pCP20 plasmid into the cells of the corrected clone and plated them on the selection plate containing chloramphenicol after 5 h of recovery, which was incubated at 30°C overnight. The next day, we cultured the positive clone in LB medium containing chloramphenicol with shaking (220 rpm) at 37°C. Subsequently, the culture was plated on an LB plate and incubated at 42°C overnight to remove the kanamycin-resistance cassette and pCP20 plasmid. For *pspA* deletion mutant construction, we amplified the kanamycin-resistance cassette flanked by FRT sites in the Keio collection to replace the *pspA* gene on the chromosome as T. Baba described (57). pBAD empty vector or pBAD-*pspA* plasmid was transformed by electroporation into *∆pspA* to generate control strain (Vector) or *pspA* rescued strain (RESC), respectively. The *pspA* PCR product was amplified from BW25993 and inserted into pBAD/Myc-His A plasmid by the Gibson Assembly method (Gibson Assembly Cloning Kit, NEB). The ampicillin-resistant cassette of pBAD/Myc-His A vector was replaced with chloramphenicol-resistance cassette by the Gibson Assembly method. The plasmids used are listed in Table 1. All primers we used are listed in Table S3. All constructs were confirmed by sequencing.

### Dormant cell ratio determination

The dormant cells of wild type and *∆pspA* were identified by time-lapse microscopy using the Flow Cell System (Bioptechs FCS2, USA). Cells from the middle stationary phase were harvested and tipped on a gel pad containing 2% (wt/vol) low melting temperature agarose in LB medium. Then, cell growth was recorded under the bright-field microscope (Zeiss Axio Observer Z1, Germany) for 5 h at 37°C. Dormant cells were defined as cells non-proliferating for 5 h during observation.

For the SYTOX green staining to distinguish with the viable cells and dead cells, the *∆pspA* cells from the middle stationary phase were washed with phosphate-buffered saline (PBS) and diluted to an OD_600_ of 0.2 in PBS with 5 µM SYTOX green reagent (Invitrogen, S7020). Then, the cells were incubated at 37°C for 15 min in the dark. After staining, the cells were washed with PBS to remove the SYTOX green reagent, resuspended in LB and collected for imaging.

### Lag time and growth rate measurement

The lag time and growth rate measurements were performed using an automated imaging and analysis system called ScanLag (58). Briefly, the wild type and *∆pspA* cultures in the middle stationary phase were diluted serially in 0.9% NaCl and plated on solid LB agar medium. The plates were placed in a ScanLag setup at 37°C, which took images of the plates automatically every 20 min. An automated image analysis application was used to extract the distribution of appearance time and growth rate of the colonies.

### Antibiotic treatment and time-kill assay

The wild type, *∆pspA*, *∆pspA* with pBAD and *∆pspA* with pBAD-*pspA* cultures in the middle stationary phase were diluted 1:20 into fresh LB medium containing the following antibiotics: 100 µg/mL ampicillin, 100 µg/mL carbenicillin, 5 µg/mL meropenem, 2 µg/mL ciprofloxacin, and 5 µg/mL levofloxacin. Then, the cells were returned to the 37°C shaker at 220 rpm. After 2, 4, 6, and 8 h of killing treatment, samples were washed with PBS (pH 7.4) to remove antibiotics, diluted serially in PBS, and then spotted on LB agar plates for overnight culturing at 37°C. Colony counting was performed on the next day. For the *pspA* rescued strain, 0.0002% arabinose was added to the cells after 5 h culturing in LB medium to induce the expression of *pspA*. For the exponential phase samples, the overnight culture of wild type and *∆pspA* were diluted 1:100 into fresh LB medium for 3 h incubation at 37°C. Then, 5 µg/mL meropenem or 2 µg/mL ciprofloxacin was directly added into the exponential phase samples to perform time-kill assays as described above.

### MIC measurement

MICs of wild type and *∆pspA* to ampicillin, carbenicillin, meropenem, ciprofloxacin, and levofloxacin were determined *in vitro* by broth microdilution according to Clinical and Laboratory Standards Institute guidelines. The inoculum size was adjusted to approximately 2 × 10^6^ CFU/mL from the middle stationary phase. After 24-h incubation in MHB medium at 37°C, the turbidity of each well was observed, and the minimum antibiotic concentration that inhibited cell growth was measured as the MIC of the antibiotic.

### Protein aggresome identification using microscopy

For protein aggresome observation under bright-field microscopy, samples were tipped on a gel pad containing 2% low melting temperature agarose in LB medium and placed for imaging under a bright-field microscope (Nikon, ECLIPSE Ti2-E). For protein aggresomes observation under fluorescence microscopy, strains labeled by HslU-TCtag*-*FlAsH or HslU-EGFP were washed with PBS and tipped on a gel pad containing 2% low melting temperature agarose in PBS medium. Then imaging was conducted by fluorescence microscopy (Zeiss Axio Observer Z1). Fluorescence illumination was provided by a solid-state laser (Coherent), 488 nm for FlAsH and EGFP. For FlAsH staining, cells containing *hslU-TCtag* were collected and washed three times with PBS and treated with 10 mM EDTA for 15 min to improve membrane permeability. Cells were then resuspended in PBS supplemented with 8 mM FlAsH-EDT2 (Invitrogen) and incubated for 30 min in the dark at 37°C. FlAsH-EDT2 was washed away before imaging.

For MOPS treatment, 40 mM MOPS was added to the LB medium of *∆pspA* cells at the beginning of culturing, and then cells with or without MOPS were cultured for 16 h to middle stationary phase at 37°C in a shaker (220 rpm). Subsequently, we observed the MOPS treated and untreated *∆pspA* cells under a bright-field microscope as described above.

For CCCP treatment, wild type and *∆pspA* cells from early stationary phase were treated with 20 µM CCCP for 1.5 h at 37°C without shaking. Then, the cells treated with CCCP were observed under a bright-field microscope as described above.

### DiBAC_4_(3) staining

Wild type and *∆pspA* cells from early stationary phase were treated with 20 µM CCCP for 1.5 h. Then, the samples were collected, washed with PBS, and diluted 1:50 into PBS supplemented with 2 µM DiBAC_4_(3). Then, the samples were incubated in the dark for 20 min at 37°C. After staining, the cells were washed with PBS and collected for imaging. The imaging was conducted by the Nikon ECLIPSE Ti2-E with a ×100 oil-immersion objective. The fluorescence of DiBAC_4_(3) signaling was detected by the LED lamp with the FITC filter.

### Time-lapse recording of antibiotic killing and cell resuscitation

Antibiotic killing and bacterial resuscitation process was recorded by time-lapse microscopy using the Flow Cell System (Bioptechs FCS2, USA). Cells from the middle stationary phase were harvested, washed with PBS, and stained with SYTOX green for 15 min. Then, cells were tipped on a gel pad containing 2% (wt/vol) low melting temperature agarose in LB medium. We injected a fresh LB medium containing 150 µg/mL ampicillin and 2.5 µM SYTOX green into the FCS2 chamber and recorded the antibiotic-killing process for 5 h at 37°C (Zeiss Axio Observer Z1, Germany). Afterward, 3 mL fresh LB medium without antibiotics was injected into the FCS2 chamber to remove the antibiotics. We recorded the resuscitation process of persister cells for 16 h at 37°C.

### RNA isolation and RNA-seq

The total RNA of wild type and *∆pspA* cells from the middle stationary phase was extracted by an RNAprep Pure Cell/Bacteria Kit (TIANGEN) according to the manufacturer’s instructions. The mRNA was purified from total RNA using probes to remove rRNA. Next, the mRNA was fragmented using divalent cations under elevated temperature in First Strand Synthesis Reaction Buffer (5×) to the desired length and reverse transcribed into first-strand cDNA. The single-strand cDNA was used for double-strand DNA synthesis, followed by end repair, dA-tailing, adaptor ligation, and PCR amplification. To preferentially select cDNA fragments of 370–420 bp in length, the library fragments were purified with AMPure XP system (Beckman Coulter, Beverly, USA). Before sequencing, the library was examined by length determination and quantitative PCR certification. These constructed libraries were then sequenced by an Illumina NovaSeq platform, and 150 bp paired-end reads were generated.

### Sequencing data analysis

Clean data (clean reads) were obtained by removing reads containing adapters, reads containing N bases, and low-quality reads from raw data. Clean reads were aligned to *E. coli* str. K-12 substr. MG1655 RefSeq GCF_000005845.2 (https://ftp.ncbi.nlm.nih.gov/genomes/all/GCF/0-00/005/845/GCF_000005845.2_ASM584v2/) using Bowtie2-2.2.3. HTSeq v0.6.1 was used to count the read numbers mapped to each gene. Then, the FPKM of each gene was calculated based on the length of the gene and read count mapped to this gene. Differential gene expression analysis was performed using the DESeq R package (1.18.0). For gene functional enrichment analysis, the DEGs were submitted to DAVID for KEGG pathway analysis.

### Cellular ATP measurement

The cellular ATP levels of wild type and *∆pspA* cells from the middle stationary phase were measured using the BacTiter-Glo Microbial Cell Viability Assay (Promega, G8231) following the manufacturer’s instructions. Luminescence was recorded using a BioTek Synergy plate reader. Simultaneously, the same samples were diluted 10 times and recorded on the same plate reader at 600 nm absorbance for the optical density measurement. The total ATP content was calculated by the luminescence and ATP standard curve. The cell number was determined through OD_600_ and standard curve. Finally, the cellular ATP concentration was calculated through normalizing total ATP content by cell number and single-cell volume.

### Glucose supplementation

In the glucose supplementation group, we added 0.5% glucose to the LB medium of *∆pspA* cells at the beginning of culturing. Then, cells with or without glucose were cultured for 16 h to the middle stationary phase at 37°C in a shaker (220 rpm). Subsequently, the glucose treated or untreated *∆pspA* cells were collected for cellular ATP measurement, protein aggresome identification, and persister counting assay as described above.

### Image processing

Image analysis was done by ImageJ software (Fiji). Outlines of cells were identified from bright-field images. The fluorescence intensity was calculated by subtracting the background fluorescence from the overall fluorescence of the bacteria cell.

### Statistical analysis

Statistical analysis was performed by using GraphPad Prism (v8.0), as described in the figure legends. The averages are shown, with error bars indicating the SD. The *P* value was calculated via two-tailed Student’s *t* test or one-way ANOVA with Sidak’s posttest for multiple comparison, with *P < 0.05, **P < 0.01, and ***P < 0.005.

## Data Availability

Raw sequencing reads of RNA-seq files from this study are available in the NCBI, Sequence Read Archive (SRA accession no. PRJNA948174).

## SUPPLEMENTAL MATERIAL

Fig. S1 aac.00937-23-s0001.docx SYTOX green staining and doubling time.

Fig. S2 aac.00937-23-s0002.docx Time-kill curves.

Fig. S3 aac.00937-23-s0003.docx Imaging of HslU-EGFP foci.

Fig. S4 aac.00937-23-s0004.docx PMF measurement after CCCP treatment.

Table S1 aac.00937-23-s0005.docx MIC values.

Table S2 aac.00937-23-s0006.xlsx Differential expressed genes.

Table S3 aac.00937-23-s0007.docx Primers.

ASM does not own the copyrights to Supplemental Material that may be linked to, or accessed through, an article. The authors have granted ASM a non-exclusive, world-wide license to publish the Supplemental Material files. Please contact the corresponding author directly for reuse.
