# Supplementary material for: The metabolic slowdown caused by the deletion of pspA accelerates protein aggregation during stationary phase facilitating antibiotic persistence
Source: Antimicrob Agents Chemother. 2024 Jan 3;68(2):e00937-23. doi: 10.1128/aac.00937-23 (PMC10848772; doi:10.1128/aac.00937-23)
Supplement: Fig. S1 — SYTOX green staining and doubling time. [file aac.00937-23-s0001.docx]

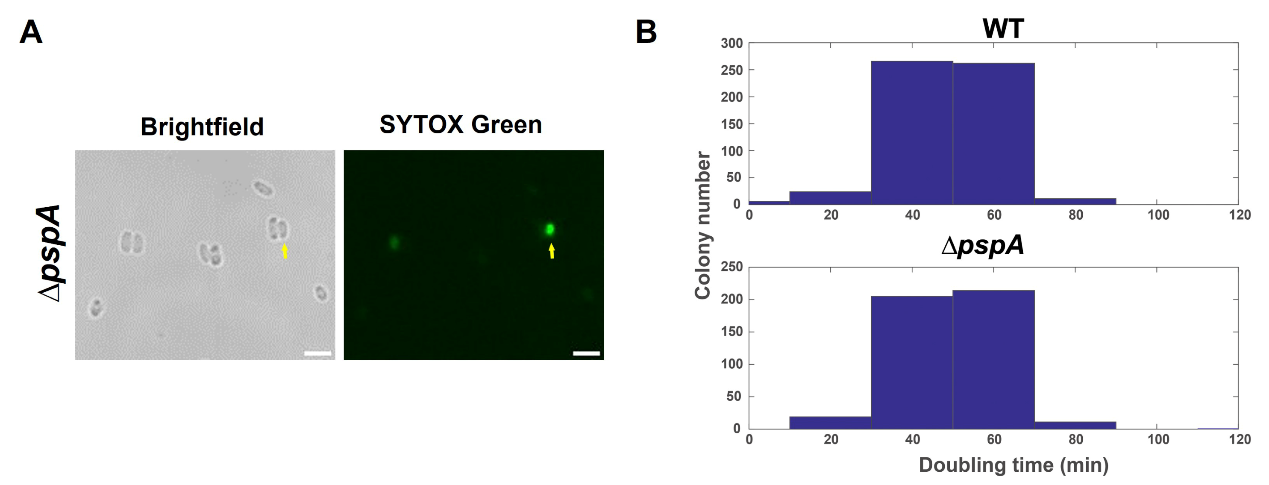


**FIG S1** (A) The SYTOX green staining image of cells shown in Fig.1A to distinguish viable cells from dead cells. The SYTOX green positive cell was pointed by the yellow arrow. Scale bar, 3 μm. (B)The doubling time of wild type and *∆pspA* cells measured by ScanLag. For wild type, the doubling time of 92.79% cells concentrated in 30-70 min, and for *∆pspA*, the doubling time of 93.11% cells concentrated in 30-70 min.
