# Supplementary material for: The metabolic slowdown caused by the deletion of pspA accelerates protein aggregation during stationary phase facilitating antibiotic persistence
Source: Antimicrob Agents Chemother. 2024 Jan 3;68(2):e00937-23. doi: 10.1128/aac.00937-23 (PMC10848772; doi:10.1128/aac.00937-23)
Supplement: Fig. S2 — Time-kill curves. [file aac.00937-23-s0002.docx]

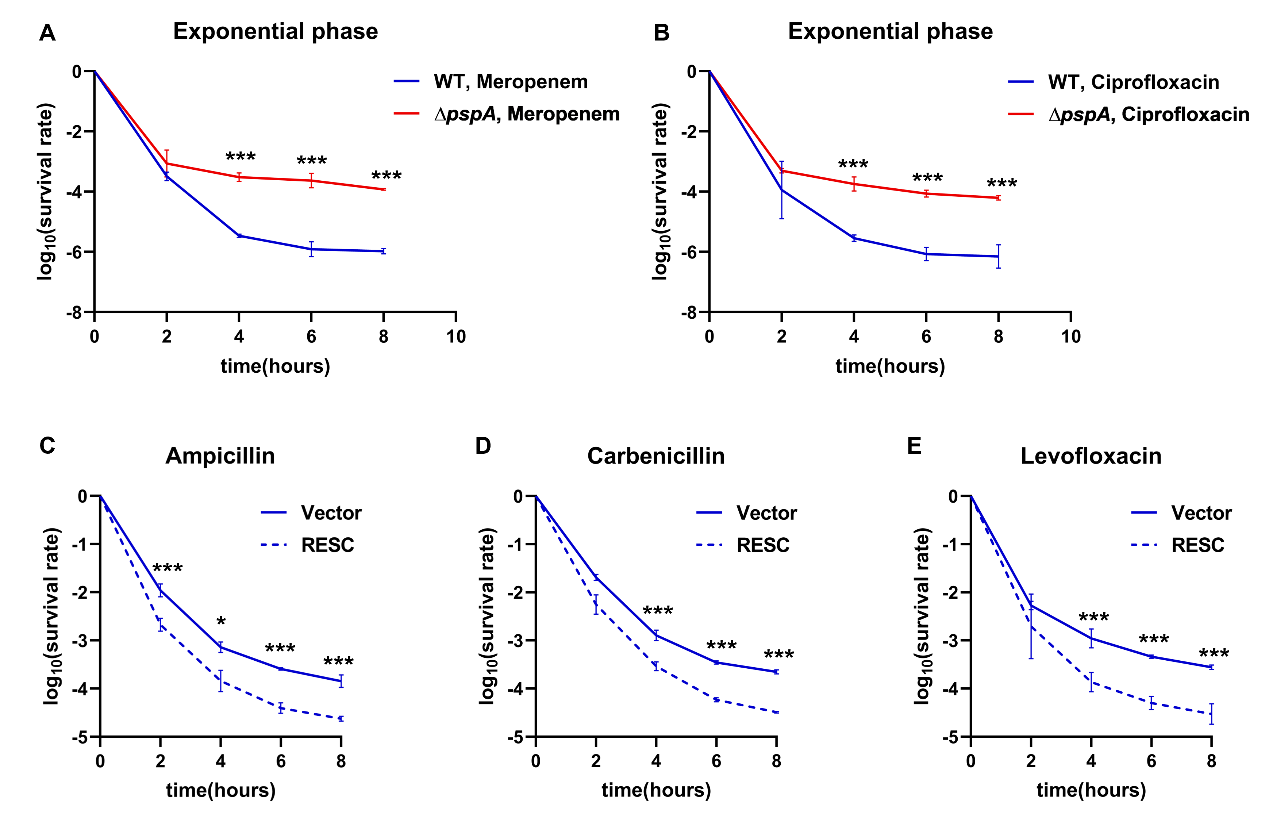


**FIG S2** Time-kill curves of wild type, *∆pspA*, *∆pspA* with pBAD (Vector) and *∆pspA* with pBAD-*pspA* (RESC). The wild type and *∆pspA* from exponential phase were treated with (A) 5 μg/ml meropenem and (B) 2 μg/ml ciprofloxacin respectively, and incubated for 8 hr at 37°C with shaking. *∆pspA* with pBAD (Vector) and *∆pspA* with pBAD-*pspA* (RESC) from middle stationary phase were diluted at a ratio of 1:20 in fresh LB medium with (C) 100 μg/ml ampicillin, (D) 100 μg/ml carbenicillin or (E) 5 μg/ml levofloxacin, and incubated for 8 hr at 37°C with shaking. The number of viable cells was counted before and after antibiotic treatment for 2 hr, 4 hr, 6 hr and 8 hr. The error bar indicates the standard deviation of at least three independent experiments. The significance was analyzed via two-tailed Student’s t test. (*, *P* < 0.05; **, *P* < 0.01; ***, *P* < 0.005).
