# Supplementary material for: The metabolic slowdown caused by the deletion of pspA accelerates protein aggregation during stationary phase facilitating antibiotic persistence
Source: Antimicrob Agents Chemother. 2024 Jan 3;68(2):e00937-23. doi: 10.1128/aac.00937-23 (PMC10848772; doi:10.1128/aac.00937-23)
Supplement: Fig. S3 — Imaging of HslU-EGFP foci. [file aac.00937-23-s0003.docx]

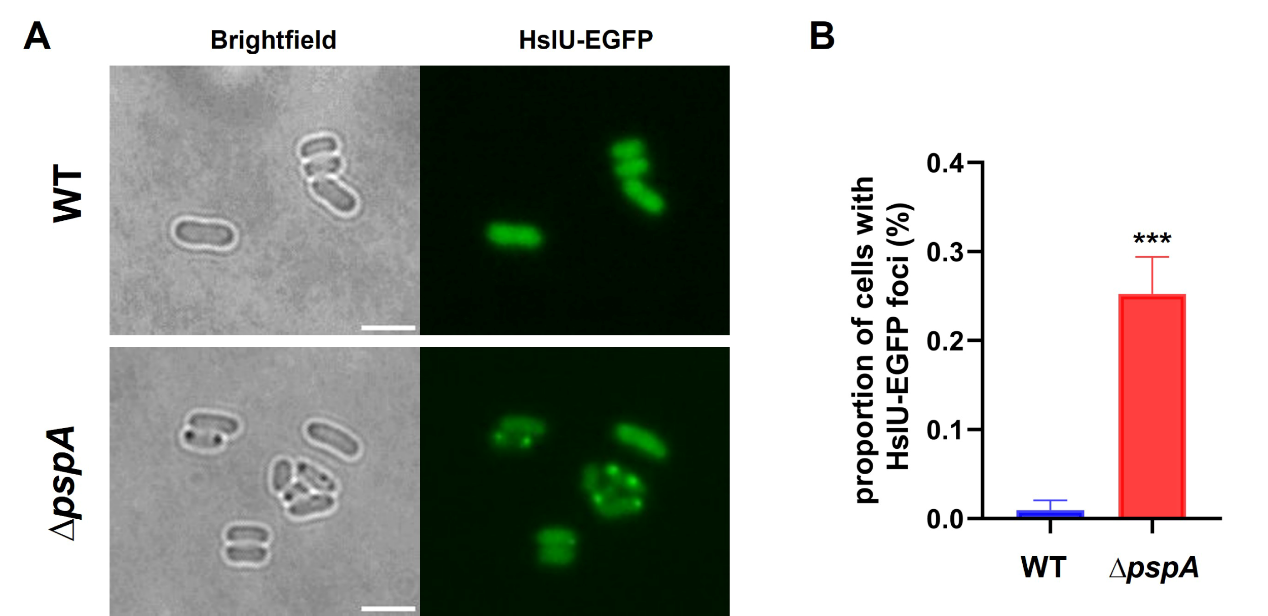


**FIG S3** (A)Bright-field and fluorescence images of HslU-EGFP labeled cells of wild type and *∆pspA*. (B)Quantification of the proportion of cells with HslU-EGFP fluorescence foci from the images. Each bar indicates the mean ± standard deviation of at least three independent experiments. The significance was analyzed via two-tailed Student's t test. *, *P* < 0.05; **, *P* < 0.01; ***, *P* < 0.005.
