# Supplementary material for: The metabolic slowdown caused by the deletion of pspA accelerates protein aggregation during stationary phase facilitating antibiotic persistence
Source: Antimicrob Agents Chemother. 2024 Jan 3;68(2):e00937-23. doi: 10.1128/aac.00937-23 (PMC10848772; doi:10.1128/aac.00937-23)
Supplement: Fig. S4 — PMF measurement after CCCP treatment. [file aac.00937-23-s0004.docx]

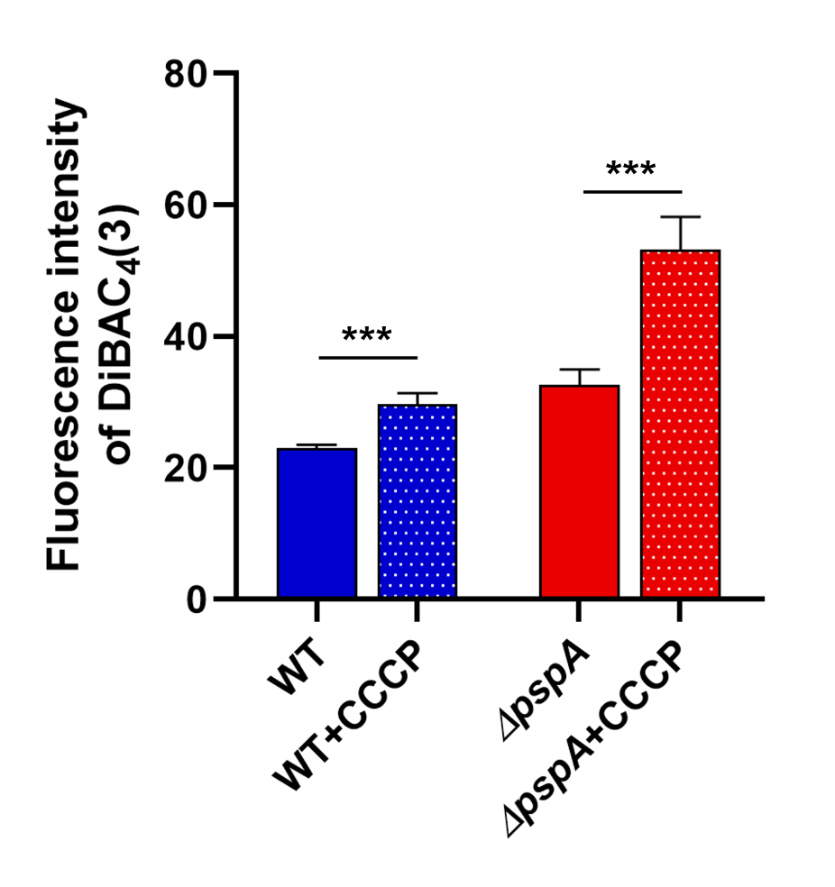


**FIG S4** The fluorescence intensity of DiBAC_4_(3) in wild type, *∆pspA,* wild type treated with CCCP and *∆pspA* treated with CCCP. Wild type and *∆pspA* cells from early stationary phase were treated with 20 μM CCCP for 1.5 h. Then the samples were stained with 2 μM DiBAC_4_(3) for 20 min at 37°C. After staining, the cells were washed with PBS and collected for imaging. Each data bar indicates the mean ± SEM of at least three independent experiments. The significance of the two data bars, indicated by a line and asterisk spanning above them, was analyzed via two-tailed Student's t test. *, *P* < 0.05; **, *P* < 0.01; ***, *P* < 0.005.
