# Supplementary material for: The metabolic slowdown caused by the deletion of pspA accelerates protein aggregation during stationary phase facilitating antibiotic persistence
Source: Antimicrob Agents Chemother. 2024 Jan 3;68(2):e00937-23. doi: 10.1128/aac.00937-23 (PMC10848772; doi:10.1128/aac.00937-23)
Supplement: Table S1 — MIC values. [file aac.00937-23-s0005.docx]

**S1 Table** Minimal inhibitory concentration (MIC) of the wild type and *∆pspA* for the antibiotics used in this work. The unchanged MIC values of wild type and *∆pspA* suggested that the enhanced survival of *∆pspA* under various antibiotic treatments was not due to resistance mechanisms.

| MIC (μg/ml) | Ampicillin | Carbenicillin | Meropenem | Ciprofloxacin | Levofloxacin |
| --- | --- | --- | --- | --- | --- |
| Wild type | 4 | 4 | 0.015 | < 0.015 | 0.03 |
| *∆pspA* | 4 | 4 | 0.015 | < 0.015 | 0.03 |
