# Supplementary material for: The metabolic slowdown caused by the deletion of pspA accelerates protein aggregation during stationary phase facilitating antibiotic persistence
Source: Antimicrob Agents Chemother. 2024 Jan 3;68(2):e00937-23. doi: 10.1128/aac.00937-23 (PMC10848772; doi:10.1128/aac.00937-23)
Supplement: Table S3 — Primers. [file aac.00937-23-s0007.docx]

**S3 Table** Primers used in this study.

| Name | Sequence (5’-3’) |
| --- | --- |
| *pspA* knockout F | cagttcagcaggacaatcctgaacgcagaaatcaagaggacaacattatgATTCCGGGGATCCGTCGACC |
| *pspA* knockout R | cggcgcgttcggacgccgcctggatgttattattgattgtcttgcttcatTGTAGGCTGGAGCTGCTTCG |
| *pspA* knockout verification F | gataaaaaattggcacgca |
| *pspA* knockout verification R | CTGCGTGCAATCCATCTTGTTC |
| hslU-egfp homology F | TGCGTTGGTGGCAGATGAAGATCTGAGCCGTTTTATCCTAggtggatccggcggttctGTGA |
| hslU-egfp homology R | TTCAGCCCCATCAAACAATGATGAAAATGATTGAACGCGAGTGTAGGCTGGAGCTGCTTCG |
| hslU-egfp verification F | GCTCGTCGTTTACACACTGT |
| hslU-egfp verification R | CGATAATTGCAGCAAAGGCGA |
| Egfp for overlap PCR F | ggtggatccggcggttctGTGAGCAAGGGCGAGGAGCTGTTC |
| Egfp for overlap PCR R | CCCGGAATTTACTTGTACAGCTCGTCCA |
| FRT cassette for overlap PCR F | ACAAGTAAATTCCGGGGATCCGTCGACC |
| FRT cassette for overlap PCR R | GTGTAGGCTGGAGCTGCTTCG |
| pBAD backbone F | TGAGTTTAAACGGTCTCCAGCTTG |
| pBAD backbone R | CATGGTTAATTCCTCCTGTTAGCC |
| pspA for pBAD F | GGCTAACAGGAGGAATTAACCATGggtattttttctcgctttgcc |
| pspA for pBAD R | CAAGCTGGAGACCGTTTAAACTCATTATTGATTGTCTTGCTTCATTTTG |
